# MAPK Activation Is Essential for *Waddlia chondrophila* Induced CXCL8 Expression in Human Epithelial Cells

**DOI:** 10.1371/journal.pone.0152193

**Published:** 2016-03-22

**Authors:** Skye Storrie, David Longbottom, Peter G. Barlow, Nick Wheelhouse

**Affiliations:** 1 Moredun Research Institute, Pentlands Science Park, Edinburgh, Midlothian, EH26 0PZ, United Kingdom; 2 School of Life, Sport and Social Sciences, Edinburgh Napier University, Sighthill Campus, Edinburgh, EH11 4BN, United Kingdom; University of the Pacific, UNITED STATES

## Abstract

**Background:**

*Waddlia chondrophila* (*W*. *chondrophila*) is an emerging agent of respiratory and reproductive disease in humans and cattle. The organism is a member of the order Chlamydiales, and shares many similarities at the genome level and in growth studies with other well-characterised zoonotic chlamydial agents, such as *Chlamydia abortus* (*C*. *abortus*). The current study investigated the growth characteristics and innate immune responses of human and ruminant epithelial cells in response to infection with *W*. *chondrophila*.

**Methods:**

Human epithelial cells (HEp2) were infected with *W*. *chondrophila* for 24h. CXCL8 release was significantly elevated in each of the cell lines by active-infection with live *W*. *chondrophila*, but not by exposure to UV-killed organisms. Inhibition of either p38 or p42/44 MAPK significantly inhibited the stimulation of CXCL8 release in each of the cell lines. To determine the pattern recognition receptor through which CXCL8 release was stimulated, wild-type HEK293 cells which express no TLR2, TLR4, NOD2 and only negligible NOD1 were infected with live organisms. A significant increase in CXCL8 was observed.

**Conclusions/Significance:**

*W*. *chondrophila* actively infects and replicates within both human and ruminant epithelial cells stimulating CXCL8 release. Release of CXCL8 is significantly inhibited by inhibition of either p38 or p42/44 MAPK indicating a role for this pathway in the innate immune response to *W*. *chondrophila* infection. *W*. *chondrophila* stimulation of CXCL8 secretion in HEK293 cells indicates that TLR2, TLR4, NOD2 and NOD1 receptors are not essential to the innate immune response to infection.

## Introduction

*Waddlia chondrophila* (*W*. *chondrophila*) is an emerging agent of respiratory and reproductive disease in humans and cattle. One of the first *Chlamydia*-related organisms to be successfully isolated, *W*. *chondrophila* was originally obtained from a case of bovine abortion in the United States in 1986 [1,2]. From a veterinary perspective, *W*. *chondrophila* has been studied exclusively as a reproductive pathogen. However, a number of human studies have indicated potential pathogenic roles in both reproductive [3,4] and respiratory conditions [5] suggesting that the organism can infect and replicate at multiple mucosal sites throughout the host. This is corroborated by in vitro studies in which the organism was shown to be capable of infecting a wide variety of human cell lines derived from a number of different lineages [6].

Pro-inflammatory cytokine and chemokine secretion, including CXCL8 production, is associated with chlamydial infection of epithelial cells leading to pathogenesis of infection. Expression of these pro-inflammatory mediators occurs through distinct signalling pathways whose stimulation is through interactions of the host cells with specific pathogen associated molecular patterns (PAMPS) [7]. Comparative studies have demonstrated distinct differences in the membrane structure [8] and the developmental cycle [9] of *W*. *chondrophila* compared to other pathogenic chlamydial species, which have been suggested to reduce the pathogenicity of the organism. Despite these observed differences, infection of ovine trophoblast cells with *W*. *chondrophila* leads to a pro-inflammatory response [10] similar to that observed with the pathogen *C*. *abortus* [11] suggesting stimulation of similar signalling pathways within the host cell.

It has been previously established that a number of human epithelial cells express CXCL8 in response to chlamydial infection, and that this response is at least partially occurring through activation of the p42/44 MAPK cascades [12]. Given the zoonotic potential of *W*. *chondrophila* as a significant emerging pathogen in humans, and the central role that CXCL8 secretion by infected epithelial cells plays during the initiation of inflammation, this study was performed in order to ascertain if infection of human epithelial cells with *W*. *chondrophila* would stimulate CXCL8 release, and to investigate the signalling pathways which may be responsible for this response.

## Materials & Methods

### Cell culture & CXCL8 analysis

HEp2 and HEK293 cells were obtained from the European Collection of Cell Cultures (ECACC, Salisbury, UK). HEp2 cells were routinely grown in Iscove’s Modified Dulbecco’s Medium (IMDM, Life Technologies, Paisley, UK) supplemented with 5% heat inactivated fetal calf serum (FCS, PAA Laboratories Ltd, Yeovil, Somerset, UK). HEK293 cells were cultured in Dulbecco’s Modified Essential Medium (DMEM, Life Technologies), with 10% FCS. CXCL8 was quantified using a commercial human IL-8 ELISA duo-set ELISA kit provided with internal standards (R & D systems).

### Experimental infections & treatments

*Waddlia chondrophila* strain ATCC VR-1470 was grown at 37°C in HEp2 cells, titrated on 8-well chamber slides (BD Falcon, Becton Dickinson, Bedford, UK) and visualised according to previously published protocols using a polyclonal antibody raised against *W*. *chondrophila* elementary bodies (a kind gift from Professor Gilbert Greub, University of Lausanne) [10]. To investigate the effect of *W*. *chondrophila* on CXCL8 release, 1 x10^5^ cells (HEp2 and HEK293) were seeded overnight and grown to sub-confluence in 48 well plates (Corning Costar, High Wycombe, United Kingdom). The cell lines were exposed to a control cell lysate (medium control) infected with *W*. *chondrophila* at an estimated multiplicity of infection (MOI) of 10 or exposed to UV-killed organisms (treated with 2MJ UV-C; MOI 10 equivalent) in their respective media containing 2% FCS as previously described [10, 11]. Supernatants and lysates for DNA extraction (see below) were harvested 24h post-infection. For experiments to determine the effects of pharmacological inhibitors, UO126 (p42/44 inhibitor), SB202190 (p38 inhibitor) (Both Invivogen, Toulouse, France), ML-130 (NOD1 inhibitor; Bio-Techne, Abingdon, UK) upon CXCL8 secretion, cells were pre-treated with inhibitors 2h prior to the addition of *W*. *chondrophila*. During experiments in which the effects of *W*. *chondrophila* replication upon CXCL-8 release were investigated, chloramphenicol (Fisher Scientific, Loughborough, UK) was added 2h post-infection. To quantify NOD1 induced CXCL8 secretion in non-infected HEK293s, cells were exposed to C12-ie-DAP for 24h (Invivogen). All treatments were performed on triplicate wells and experiments conducted on three separate occasions.

### Quantification of *W*. *chondrophila* replication

DNA was isolated using the DNeasy^®^ Blood and Tissue kit (Qiagen, Crawley, UK). The monolayers were lysed directly in 200μl AL buffer (supplied with DNeasy^®^ Blood and Tissue kit). The cell lysate and pellet resulting from centrifugation of the medium were combined and thoroughly mixed. The combined lysate was mixed with 200μl PBS and 20μl Proteinase K prior to incubation at 55°C for 10 minutes. Absolute ethanol (200μl) was added to each sample and DNA extracted according to the manufacturer’s instructions.

To quantify the replication of the organism a pan-*Chlamydiales* qPCR targeting the 16S rRNA gene was performed [10] using the forward primer panCh16F2 (5’-CCGCCAACACTGGGACT-3’), the reverse primer panCh16R2 (5’-GGAGTTAGCCGGTGCTTCTTTAC-3’) and the probe panCh16S (5’-FAM-CTACGGGAGGCTGCAGTCGAGAATC-BHQ1-3’) [13]. Assays were performed in a total volume of 20 μl, using the Quanta Toughmix Low ROX (Quanta BioSciences, Inc., Gaithersburg, USA), 0.1 μM primer (Exiqon, Vedbaek, Denmark), a 0.1 μM probe (Integrated DNA Technologies, Iowa, USA), molecular-biology-grade water (Promega, Southampton, UK), and 1 μl DNA. The cycling conditions were 3 min at 95°C, 50 cycles of 15 s at 95°C, 15 s at 67°C and 15 s at 72°C. Detection was performed on an ABI 7500 (Life Technologies). Molecular grade water was used as a negative PCR control. Quantification was achieved using a standard curve derived using a recombinant plasmid control, as previously described [10].

### Statistics

Data were analysed by one way-way ANOVA. Comparisons between individual treatments were made using Fisher’s least significant difference test. All analyses were performed using Genstat Version 11.

## Results

### CXCL8 response of epithelial cells to infection

**Fig 1:**
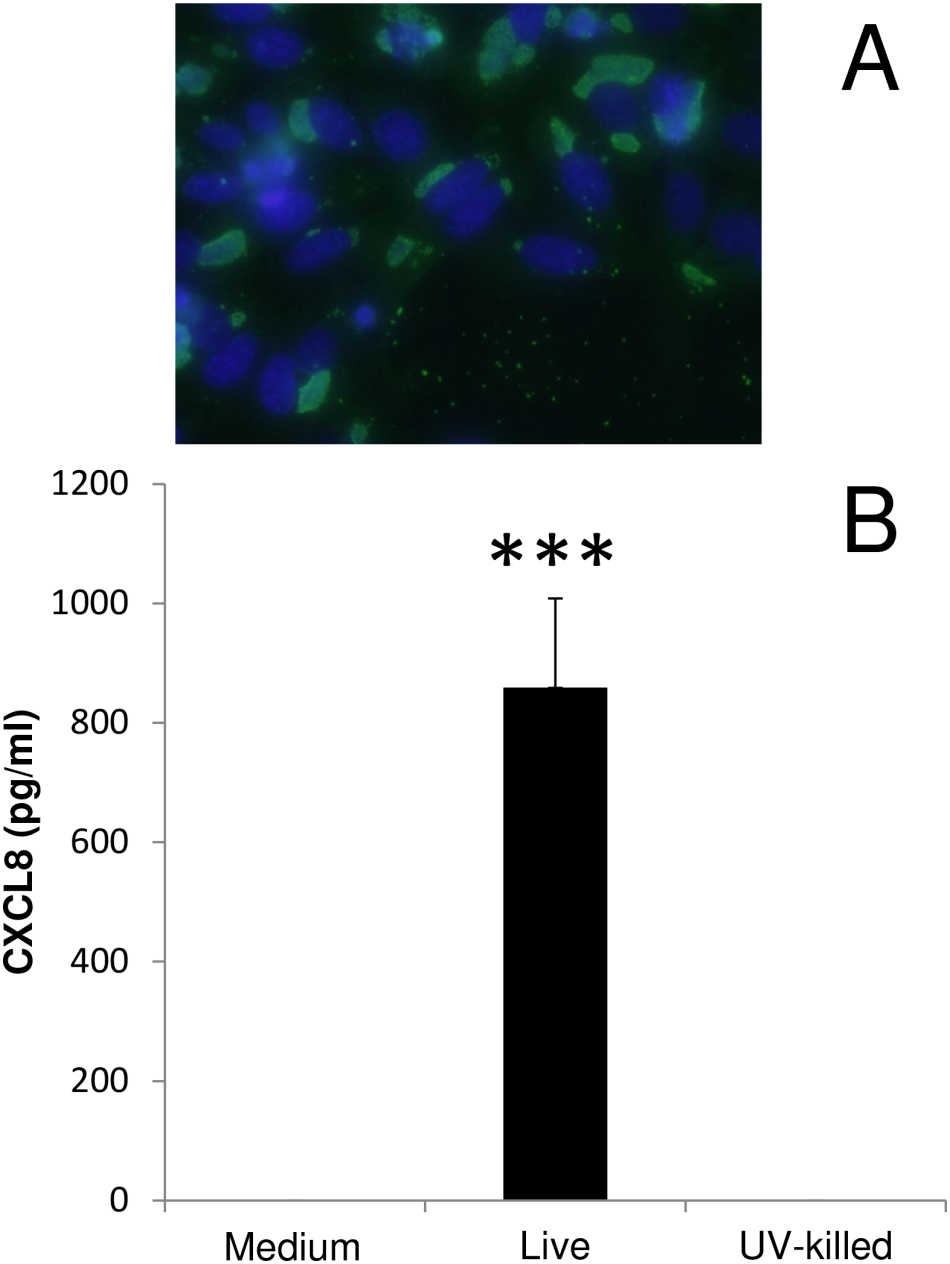
Bacterial growth and CXCL8 release in HEp2 cells. A) Fluorescent micrographs demonstrating the infectivity of *W*. *chondrophila* at an apparent MOI of 10 at 24h post-infection. *W*. *chondrophila* inclusions are labelled green using an anti-*Waddlia* rabbit polyclonal antisera and FITC anti-rabbit secondary antibody, host cell nuclei are stained in blue (DAPI). The scale bars correspond to 100μm. B) *W*. *chondrophila* infection induces CXCL8 production at 24 hours pi in HEp2 cells. Cells were to medium containing uninfected cell lysate (Med), *W*. *chondrophila* (Live) or UV-killed organisms (MOI 10) for 24h. Statistically-significant differences relative to medium control are indicated by ***P<0.001. Data were analysed by one way ANOVA followed by Fishers LSD test.

Chlamydial infection of epithelial cells initiates an influx of neutrophils to the site of infection. Therefore, the effects of *W*. *chondrophila* infection upon the expression of the chemokine CXCL8 was investigated. Infection with live *W*. *chondrophila* at an MOI 10 (visualised in Fig 1A) led to a significant increase in CXCL8 (0 vs 859 ± 149 pg/ml; p<0.001) (Fig 1B). However, exposure of the cells to either the medium control or UV-killed organisms (MOI 10) failed to elicit a response in CXCL8 expression, in agreement with our previous findings in ovine trophoblasts.

### MAPK phosphorylation and CXCL8 release

**Fig 2:**
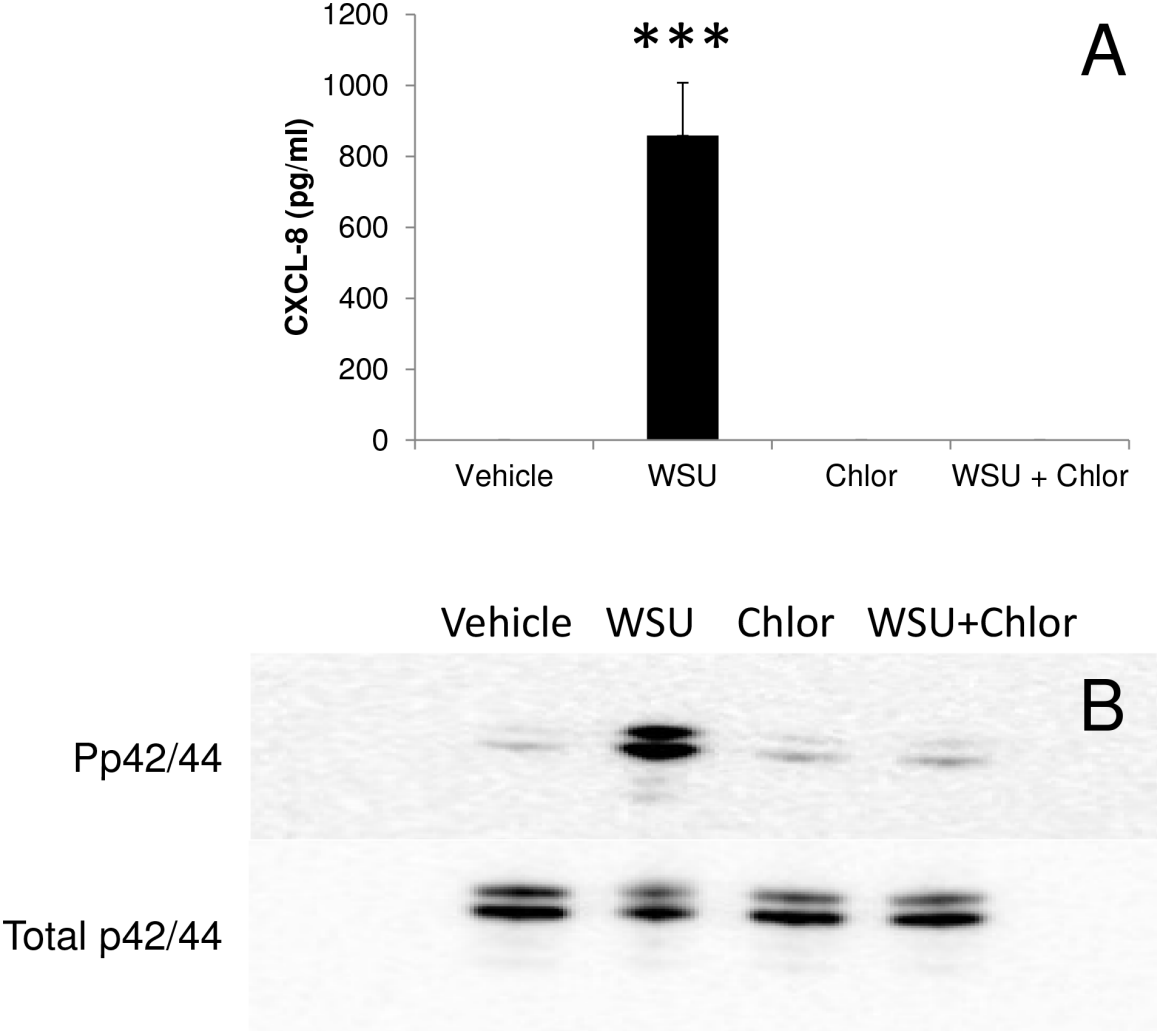
Effects of chloramphenicol upon the response of HEp2 cells to infection. Cells were infected with *W*. *chondrophila* (WSU) for 2 hours before the addition of chloramphenicol. Supernatants and protein extracts were obtained 24 hours post-infection. A) Treatment with chloramphenicol inhibits CXCL8 secretion after *W*. *chondrophila* infection. Statistically-significant differences relative to medium control are indicated by ***P<0.001. Data were analysed by one way ANOVA followed by Fishers LSD test. B) *W*. *chondrophila* infection stimulates phosphorylation of p42/44 MAPK but there is no phosphorylation in the presence of chloramphenicol.

It has been demonstrated that activation of MAPK pathways are important in chlamydial growth and that pharmacological inhibition of these pathways can inhibit active replication of the organism in the host cell. In order to determine whether active bacterial growth is essential for CXCL8 production in *W*. *chondrophila* infected cells, HEp2 cells were treated with the antibiotic chloramphenicol 2 hours post-infection with live *W*. *chondrophila* (MOI 10). 24 hours after the addition of chloramphenicol a significant reduction in bacterial genome copy numbers was observed (1.83 x 10^8^ ± 3.89 x 10^7^ vehicle vs 3.95 x 10^5^ ± 9.70 x 10^4^ treated; p<0.001). Supernatants from these cells were also collected at 24 hours p.i. and CXCL8 quantified by ELISA. Consistent with the hypothesis that active replication of the bacteria is an essential requirement for CXCL8 release, chloramphenicol treatment also led to a significant reduction in CXCL8 production in *W*.*chondrophila* infected HEp2 cells (1365 ± 171 pg/ml control vs 35 ± 18 pg/ml chloramphenicol treated; p<0.001) (Fig 2A). Moreover, the chloramphenicol treatment was also associated with an absence of p42/44 phosphorylation indicating that active replication of the organism is required for stimulation of the p42/44 MAPK cascade (Fig 2B).

**Fig 3:**
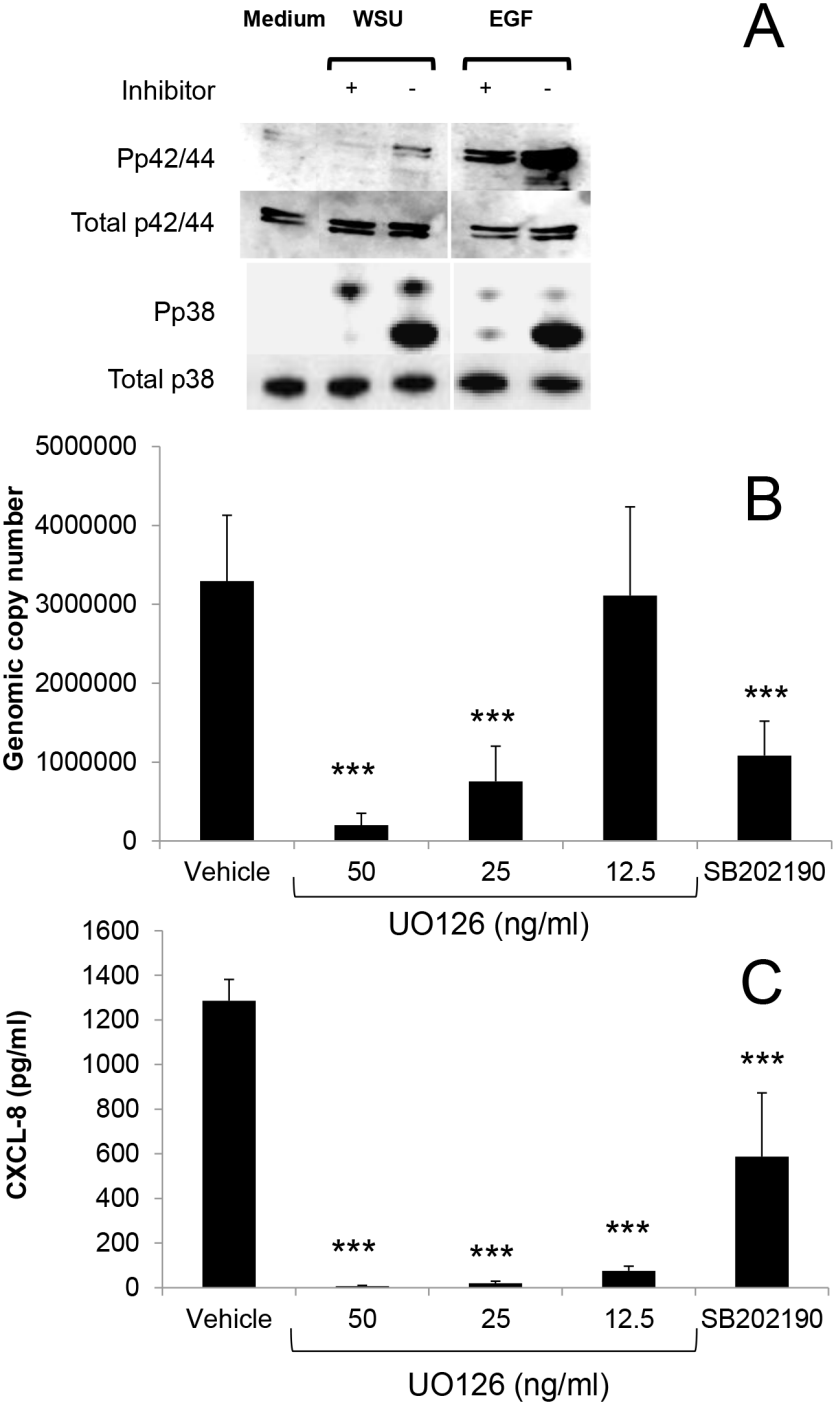
The effects of MAPK inhibitors upon *W*. *chondrophila* replication and CXCL8 secretion in HEp2 cells. Cells were pre-treated for 2h prior to infection with *W*. *chondrophila* (MOI 10). A) Western blot demonstrating induction of p42/44 and p38 MAPK by *W*. *chondrophila* and inhibition by the specific inhibitors UO126 (p42/44 MAPK) and SB202190 (p38 MAPK). As a positive control for both p42/44 and p38 phosphorylation cells were exposed to EGF for 5 mins. B) and C) The effects of increasing concentrations of the p42/44 MAPK inhibitor UO126 on bacterial replication and CXCL8 release respectively. Statistically-significant reductions in CXCL8 relative to infected vehicle control cells are indicated by ***P<0.001. Data were analysed by one way ANOVA followed by Fishers LSD test.

Induction of CXCL8 expression in *Chlamydia* infected epithelial cells has been shown to be dependent upon MAPK phosphorylation. Initially experiments were carried out to determine whether infection of HEp2 cells would result in phosphorylation of p38 and p42/44 MAPK. Uninfected and *W*. *chondrophila* infected HEp2 cells were lysed 24 hours after infection and total protein extracted for analysis by immunoblotting. An increase in both phosphorylated p42/44 and p38 compared to control cells could be observed during infection (Fig 3A) and by treatment with EGF which acted as a positive control. Whereas pre-treatment with either UO126 (12.5 μM) or SB202190 (20 μM) prior to infection successfully inhibited the EGF and *W*. *chondrophila* induced phosphorylation of p42/44 and p38 MAPK. The effects of MAPK inhibitors on bacterial replication and CXCL8 release was determined in HEp2 cells and are summarised in Fig 3. Treatment with either 50 or 25 μM UO126 significantly decreased both *W*. *chondrophila* replication (3.41 x 10^6^ ± 7.80 x 10^5^ GCN/well infected vehicle vs 2.02 x 10^5^ ± 1.5 x 10^5^ 50 μM; p<0.001 and 7.56 x 10^5^ ± 4.5 x 10^5^ GCN/well 25μM; p = 0.007) (Fig 3B) and CXCL8 release (1286 ± 95 pg/ml control vs 7 ± 4 pg/ml 50 μM and 19 ± 10; both p<0.001) (Fig 3B). At a concentration of 12.5μM, UO126 was found to have no significant inhibitory effect on bacterial replication (3.41 x 10^6^ ± 7.80 x 10^5^ GCN/well vs 3.11 x 10^6^ ± 1.13 x 10^6^ GCN/well) (Fig 3C) however it did maintain an inhibitory effect upon CXCL8 at this concentration (1286 ± 95 pg/ml control vs 76 ± 21 pg/ml UO126; p<0.001) (Fig 3C).

While UO126 pre-treatment conferred a near total inhibition of CXCL8 without affecting bacterial replication, SB202190 was less effective. SB202190 treatment significantly inhibited CXCL8 secretion by 54% (1286 ± 95 pg/ml control vs 587 ± 286 pg/ml SB202190; p = 0.006) (Fig 3B), however this was accompanied by a 68% inhibition of replication (20 μM) (3.41 x 10^6^ ± 7.80 x 10^5^ GCN/well control vs 1.08 x 10^6^ ± 0.44 x 10^6^ GCN/well SB202190; p = 0.008), suggesting the inhibitory effects upon CXCL8 release were primarily dependent upon its effects on bacterial replication (Fig 3C).

### Effect of *W*. *chondrophila* infection upon CXCL8 release in HEK293 cells

**Fig 4:**
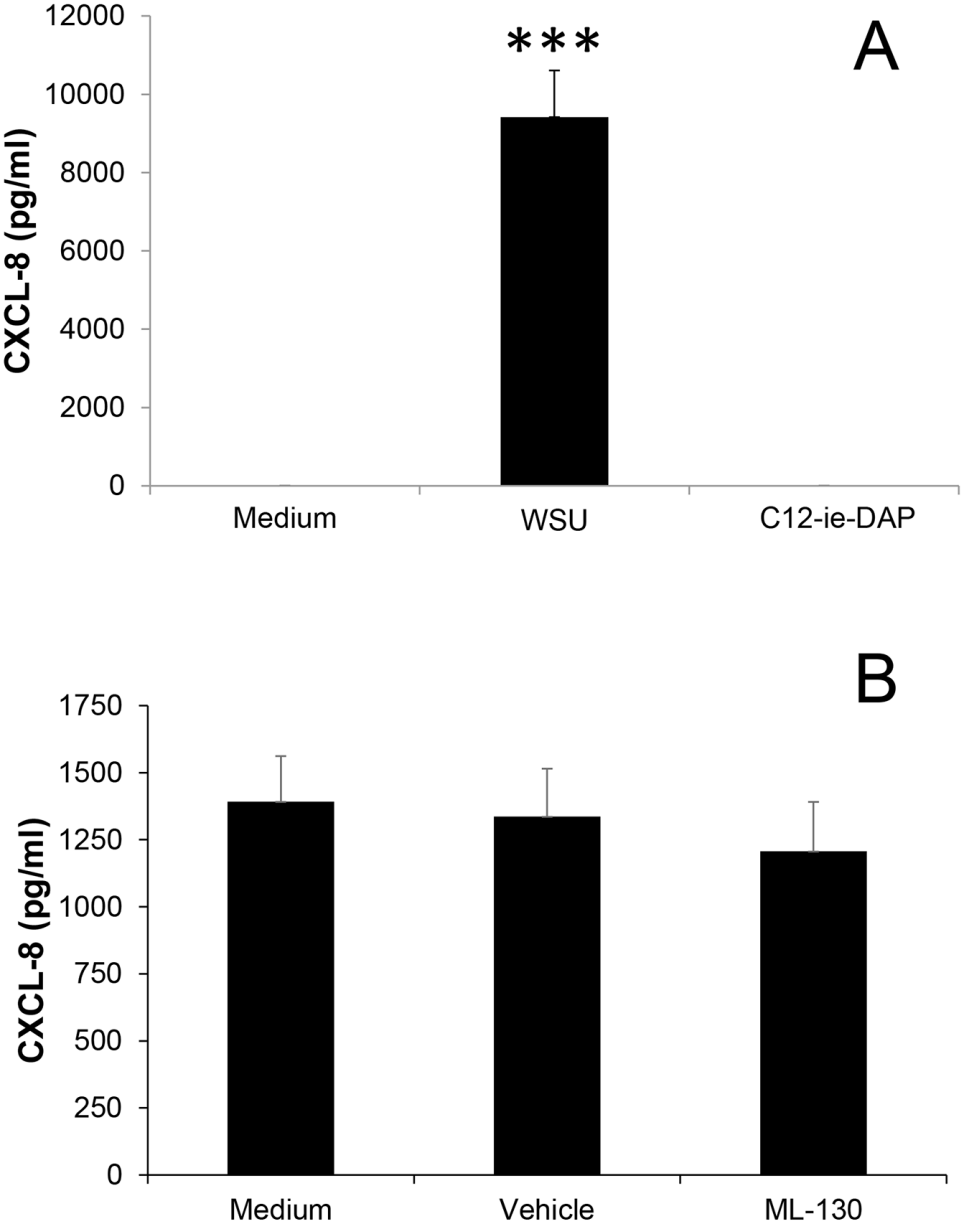
A) The effect of *W*. *chondrophila* infection or exposure the NOD-1 synthetic agonist C12-ie-DAP for 24h upon CXCL8 in HEK293 cells. B) No effect of inhibition of NOD-1 upon CXCL8 secretion in *W*. *chondrophila* infected HEp2 cells using the specific inhibitor ML-130 (1μM). Statistically-significant reduction in CXCL8 relative to infected vehicle control cells are indicated by ***P<0.001. Data were analysed by one way ANOVA followed by Fishers LSD test.

Both TLR2 and NOD-1 pattern recognition receptors have been demonstrated to be important in cytokine and chemokine release by *Chlamydia* infected epithelial cells. HEK293 cells do not express a number of pattern recognition receptors including TLR2, TLR4 and NOD2 and only negligible levels of NOD1. To initially determine whether the NOD1 activity of wild-type HEK293 cells was sufficient to elicit a response in CXCL8, cells were exposed to the NOD1 specific ligand C12-ie-DAP for 24 hours. Uninfected HEK293 cells released negligible amounts of CXCL8, however, there was no significant response in terms of CXCL8 release from C12-ie-DAP treated cells. Conversely, a significant increase in CXCL8 release was observed in cells infected with *W*. *chondrophila* (Fig 4A).

In order to further elucidate the role of NOD-1 during *W*. *chondrophila* infection, HEp2 cells were pre-treated with the NOD1-specific inhibitor ML-130 (1μM) prior to *W*. *chondrophila* infection. No significant inhibitory effects on CXCL8 release were observed, again suggesting that NOD1 is not involved in the CXCL8 response to *W*. *chondrophila* infection (Fig 4B).

## Discussion

*Waddlia chondrophila* is considered a potential zoonotic abortifacient agent and has now been identified in respiratory tract infections of human patients [5] and the placentas of aborted foetuses in both cattle [2] and humans [10]. Pathogenesis of chlamydial infections is associated with the secretion of chemokines from infected epithelial cells and the concomitant influx of inflammatory cells, including neutrophils, to the site of infection. Central to the recruitment of these neutrophils to the site of infection is the secretion of the chemokine CXCL8 by host cells. The results from the current study clearly demonstrate that *W*. *chondrophila* infection also leads to an increase in CXCL8 release from human epithelial cells, which is consistent with its role as an emerging human pathogen.

The MAPK signalling pathways have been shown to be important for CXCL8 release in cells infected with *Chlamydia* species [12]. However, *W*. *chondrophila* demonstrates differences both at the genomic level [14], membrane structure [8] and in terms of its intracellular life-cycle compared to its more recognised relatives within the *Chlamydiaceae*, and it is not known if these differences are reflected in the activation of signalling pathways responsible for CXCL8 release. During this study *W*. *chondrophila* infection significantly increased phosphorylation of both p38 and p42/44 MAPK pathways, and this activation was shown to be inhibited by treatment with specific inhibitors. Furthermore, chemical inhibition of the p42/44 and p38 MAPK pathways led to a significant reduction in CXCL8 production in *W*. *chondrophila* infected cells. This indicated that both the p42/44 and p38 MAPK pathways were required for the upregulation of CXCL8 secretion in response to *W*. *chondrophila* infection. The demonstration that p42/44 MAPK is required for the CXCL8 response of infected epithelial cells is consistent with previous studies which have demonstrated an essential requirement for this pathway in CXCL8 production in response to *C*. *trachomatis* infection ([12,15,16]. While the role of the p42/44 MAPK cascade in *Chlamydia* induced CXCL8 expression is established, the role of the p38 MAPK pathway is less clearly defined. Several studies have suggested a direct role for the pathway for both *C*. *pneumoniae* [17] and *C*. *trachomatis* [16]. However, a previous study suggested that its activation was not essential to CXCL8 production in *C*. *trachomatis*-infected HeLa cells [12].

It has been observed that chlamydial growth can be dependent upon MAPK signalling, and therefore may indirectly lead to a decrease in CXCL8 release in *Chlamydia*-infected cells. Therefore, we conducted a study to determine whether the effects of the MAPK inhibitors upon CXCL8 release was specific, or an indirect consequence of a decrease in intracellular replication. The effects of growth inhibition of the organism were studied by the use of the antibiotic chloramphenicol post-infection. Using various doses of MAPK inhibitors, the p42/44 MAPK inhibition of CXCL8 release could be observed at concentrations which appeared to have no effects on bacterial replication. However, the effect of p38 MAPK inhibition appeared to be indirect, acting solely through inhibition of bacterial growth, which is again in similarity with studies in *C*. *trachomatis* infected cells [18].

Consistent with our previous observations in ovine trophoblasts [10], only active infection, but not exposure to UV-killed organisms, stimulated CXCL8 release. This suggests that any innate immune response from the host cell is directed through cytosolic, rather than cell surface, pattern recognition receptors. Both intracellular TLR2 and the NOD1 intracellular pattern recognition receptor, which detects specific motifs in bacterial peptidoglycan, have been implicated in the induction of a CXCL8 response to chlamydial infection [7]. Recently a peptidoglycan-like structure was isolated from *W*. *chondrophila* and shown to stimulate an NF-kB reporter construct in NOD1 and NOD2 overexpressing but not wild-type HEK293 cells [19]. To determine if these pattern recognition receptors could play a role in *W*. *chondrophila*-induced CXCL8 secretion and MAPK activation, wild-type HEK293 cells, which do not express TLR2 or NOD2, and only expresses low levels of NOD1, were infected with *W*. *chondrophila*. However, infection with the organism led to the secretion of relatively high concentrations of CXCL8. This clearly demonstrates that *W*. *chondrophila* induction of CXCL8 is independent of TLR2 and NOD2. Furthermore, exposure of the cells to the NOD1 agonist, C12-ie-DAP, also failed to induce measurable CXCL8, indicating that it is unlikely that stimulation of the low levels of NOD1 in this cell line could be responsible for the very high levels of CXCL8 expression observed. Consistent with these observations that NOD1 activation is not an absolute requirement for CXCL8 release after *W*. *chondrophila* infection, incubation of *W*. *chondrophila* infected HEp2 cells with the NOD1 inhibitor ML-130 also failed to exert any inhibitory effects upon CXCL8 release. While NOD1 has been implicated in CXCL8 expression after chlamydial infection *in vitro*, it was shown that the siRNA knockdown of NOD1 expression in HeLa cells only resulted in a partial inhibition of CXCL8 secretion [7]. Furthermore in NOD1 knock-out mice, cytokine expression and pathology were the same after *Chlamydia* infection as wild-type mice demonstrating that NOD1 is not essential to elicit an innate immune response [20]. This suggests a level of redundancy in pattern recognition and that multiple pathways elicit the recognition of host cells to *Chlamydia* infection. Given the similarities in intracellular signalling and cytokine expression observed in the current study the results from the current study cannot discount the possibility that similar pathways are employed in the recognition of both *W*. *chondrophila* and *Chlamydia* species by host cells. These observations are not unique to chlamydial species and it has been demonstrated that the CXCL8 response to infection with other intracellular bacteria can occur independently of NOD1 and TLR2. High levels of CXCL8 secretion have also been observed in wild-type HEK293 cells infected with the pathogen *Burkholderia pseudomalleii* [21], even after the knock-down of basal NOD1 in the HEK293 cells. Through genetic manipulation of the organism it was demonstrated that a functional Type III secretion system was essential for CXCL8 release [21], however to date the specific pattern recognition receptor also remains to be identified.

The results of the current study demonstrate that the host p42/44 MAPK signalling pathway is stimulated during active *W*. *chondrophila* infection and induces the inflammatory mediator CXCL8. The results also demonstrate that CXCL8 expression is induced in the absence of TLR2 and NOD1 however, the pattern recognition receptor responsible remains to be elucidated. Characterizing how *W*. *chondrophila* infection stimulates the host cell response is particularly important for understanding the mechanism by which the organism facilitates its intracellular development, and also how the host cell responds to infection.
